# Developing a new predictive index for anastomotic leak following the anastomosis of esophageal atresia: preliminary results from a single centre

**DOI:** 10.1186/s13019-022-01878-8

**Published:** 2022-05-28

**Authors:** Song-Ming Hong, Qiang Chen, Hua Cao, Jun-Jie Hong, Jin-Xi Huang

**Affiliations:** 1 Department of Cardiothoracic Surgery, Fujian Branch of Shanghai Children’s Medical Center, Fuzhou City, China; 2 Fujian Children’s Hospital, 966 Hengyu Road, Fuzhou City, Fujian Province China; 3 Fujian Maternity and Child Health Hospital, Affiliated Hospital of Fujian Medical University, Fuzhou City, China; 4 Fujian Key Laboratory of Women and Children’s Critical Diseases Research, Fujian Maternity and Child Health Hospital, Fuzhou City, China

**Keywords:** Esophageal atresia, Anastomotic leak, Atresia gap length, Birth weight

## Abstract

**Background:**

The aim of this study was to determine a predictive index for the risk of anastomotic leak following esophageal atresia anastomosis,

**Methods:**

This article reviewed the clinical data of 74 children with esophageal atresia in Fujian Children's hospital. The risk factors for anastomotic leak were analysed, and a new predictive index was proposed.

**Results:**

The incidence of anastomotic leak was 29.7% after anastomosis in 74 children with esophageal atresia. Birth weight and gap length were risk factors for anastomotic leak. Logistic regression analysis showed that birth weight (Wald 2 = 4.528, *P* = 0.033, OR = 0.273) was a protective factor for anastomotic leak, whereas gap length (Wald 2 = 7.057, *P* = 0.008, OR = 2.388) was a risk factor for anastomotic leak. The ratio of gap length to birth weight had a positive predictive effect on the occurrence of anastomotic leak (AUC = 0.732, *P* = 0.002).

**Conclusion:**

Birth weight and gap length are important predictors of anastomotic leak in esophageal atresia. Measurement of the ratio of gap length to birth weight is a helpful predictive index for anastomotic leak following the anastomosis of esophageal atresia.

## Background

Esophageal atresia (EA) is a congenital disruption of esophageal continuity with or without tracheal fistula (TEF). Up to 55% of affected infants may have other congenital malformations, especially complicated congenital heart disease (CHD), which is an important factor in poor prognosis [1]. Great progress has been made in the treatment of EA in the past 20 years. The overall survival rate is more than 90%, and thoracoscopic surgery has gradually replaced open surgery as the main surgical procedure [2].

Anastomotic leak is defined as esophageal rupture caused by poor anastomotic healing after esophageal reconstruction surgery, which is the most common complication after esophageal surgery and the main cause of death [2]. Anastomotic leak is still a serious complication of EA and has an important influence on the prognosis and the quality of life of affected infants [3, 4]. We hope to propose high-risk factors for esophageal anastomotic leak and find new risk assessment indicators.

## Method

The study was approved by the ethics committee of our hospital and adhered to the tenets of the Declaration of Helsinki. All participants gave written informed consent to use their infant’s data for this study after being educated about the nature of the study, potential risks and how their data may be used. Patient and Public Involvement was not implemented in the design, conduct, reporting, or dissemination of our research.

### Patient and public involvement

This study was conducted retrospectively in a provincial hospital along the southeast coast of China. From January 2016 to June 2020, we collected 74 infants with type III EA, including 51 males and 23 females. The inclusion criteria were infants with complete clinical data after successful surgical treatment. All infants were operated on by the same general thoracic surgeon. In this study, children who were underwent staged surgery (stage I TEF resection and stage II anastomosis after natural esophageal extension) were excluded.

Type III esophageal atresia is the most common clinical type in the classification of EA. The classification of EA classification comes from the Vgot classification. The 74 patients included in this study all had type IIIb esophageal atresia (according to the Vogt classification).

### Selection of operative methods

For the selection of surgical methods, we believe that the criteria for the exclusion of thoracoscopic surgery are the cases with birth weight < 1500 g, unable to stabilize physiological parameters (circulatory failure and respiratory failure) or major cardiac abnormalities [5, 6]. For these children, we tend to choose open surgery.

### Data collection

General data were collected through the medical record system, including sex, birth weight, gestational age, preoperative mechanical ventilation, and CHD status. The albumin data came from preoperative blood tests. The length of the EA defect was determined by measuring the distance between the two ends of the oesophagus after esophageal disconnection. The operative time, the amount of blood loss and the number of anastomotic lines were obtained by summarizing the operative records. A diagnosis of anastomotic leak was confirmed by the presence of contrast leakage on esophageal contrast studies. Preoperative mechanical ventilation was defined as ventilator assistance required before surgery due to the following: respiratory failure caused by tracheesophageal fistula, gastresophageal reflux and gastrointestinal flatulence.

### Data analysis

The data were analysed using SPSS Statistics, version 22.0 software (Armonk, NY; IBM Corp.). Univariate analysis was used for risk factors. ANOVA was used for continuous variables, and the chi-square test was used for discontinuous variables. Binomial regression analysis was used for logistic regression analysis, and odds ratios (ORs) and 95% confidence intervals (95% CIs) were used to indicate associations. A *P* value < 0.05 was defined as statistically significant.

## Results

Table 1 lists the general characteristics of the infants. There were 51 male and 23 female infants. The average gestational age of the infants was 38.54 ± 1.81 weeks, and the average birth weight was 2.82 ± 0.52 kg. Seventeen infants had CHD preoperatively, and 5 were intubated preoperatively. Among them, 43 patients underwent thoracoscopic surgery, and 31 patients underwent open surgery. The incidence of anastomotic leak was 29.7% (22 in 74). In our study, 17 children were complicated with CHD (5 ventricular septal defects, 7 atrial septal defects, 4 patent ductus arteriosus and 1 tetralogy of Fallot), and the study showed that CHD did not increase the risk of esophageal anastomotic leak (*P* = 0.721).

**Table 1:** General characteristics of the infants

Characteristics	Anastomotic leak	Non-anastomotic leak	Overall
Number and Gender (F/M)	22 (15/7)	52 (36/16)	74 (51/23)
Gestational age (weeks)	38.23 ± 2.20	38.67 ± 1.62	38.54 ± 1.81
Birth weight (kg)	2.61 ± 0.58	2.91 ± 0.47	2.82 ± 0.52
*Operation*			
Thoracoscope	12	31	43
Open	10	21	31
Congenital heart disease	11	6	17
Mechanical ventilation	2	3	5

Table 2 shows the univariate results for factors influencing anastomotic leak including the following: preoperative albumin (F = 4.833, *P* = 0.031), birth weight (F = 5.427, *P* = 0.023) and length of EA defect (F = 8.08, *P* = 0.006). Factors that did not influence anastomotic leak formation included the following: operation time, blood loss, and stitch number required for anastomosis.

**Table 2:** The differences of the risk factors between two groups

Factors		Anastomotic leak	Non-anastomotic leak	F/χ^2^	*p* value
Gender	M	15	36	0.008	0.568
	F	7	16		
Combined with CHD	Yes	7	10	0.524	0.377
	No	20	37		
Operation	Open	12	31	0.163	0.440
	VATS	10	21		
Gap length (cm)		1.98 ± 1.08	1.33 ± 0.82	8.080	0.006
Birth weight (kg)		2.61 ± 0.58	2.91 ± 0.47	5.427	0.023
Albumin (g/L)		32.5 ± 3.5	34.4 ± 3.2	4.833	0.031
Surgery time (min)		143.8 ± 30.9	147.2 ± 26.4	0.223	0.638
Blood lost (ml)		32.1 ± 12.5	28.5 ± 12.3	1.317	0.255
Anastomotic stitches number	6	8	21	0.622	0.733
	7	7	12		
	8	7	19		

*VATS* video-assisted thoracoscopic surgery; *CHD* Congenital heart disease

Table 3 shows the results of logistic regression analysis. Birth weight (Wald 2 = 4.528, *P* = 0.033, OR = 0.273) was a protective factor against anastomotic leak, while gap length (Wald 2 = 7.057, *P* = 0.008, OR = 2.388) was a risk factor for anastomotic leak.

**Table 3:** The results of logistic regression analysis

Factors	B value	Wals	*p* value	OR (95% CI)
Gap length (cm)	0.871	7.057	0.008	2.388 (1.256–4.540)
Birth weight (kg)	− 1.299	4.528	0.033	0.273 (0.082–0.903)
Albumin (g/L)	− 0.68	0.095	0.471	0.471 (0.776–1.124)
Constant	3.603			

**Fig. 1:**
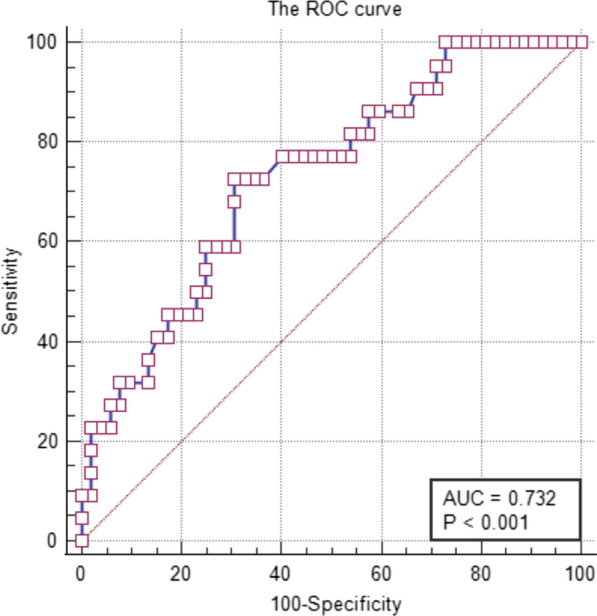
Receiver Operating Characteristic (ROC) curve

Table 4 shows that the ratio of gap length to birth weight in the anastomotic leak group was significantly higher than that in the no-nanastomotic leak group (0.71 ± 0.49 vs. 0.47 ± 0.32, *P* = 0.012). Using the receiver operating characteristic (ROC) analysis method, the area under the curve (AUC) of this indicator was found to be 0.732 (Fig. 1), which means that the ratio of gap length to birth weight had a good predictive effect on the occurrence of anastomotic leak.

**Table 4:** The data and results of ROC (Receiver Operating Characteristic) curve

AUC	*p* value	95% CI	
		Low-limit value	Up-limit value
0.732	0.002	0.61	0.853

## Discussion

EA is one of the most dangerous diseases in paediatric surgery. Improvements in surgical techniques and postoperative care have improved the survival rate for EA [7]. Despite esophageal anastomosis, infants often face severe infection and even death caused by anastomotic leak. In the past, most of these patients were successfully treated conservatively with observation, drainage and the use of antibiotics. In 1990, MacKinnon was the first to propose that EA anastomotic leak was directly related to anastomotic tension [8]. Anastomotic leak is a serious complication after EA, which can lead to serious chest infection and even the possibility of refractory stenosis [9].

Possible factors associated with its onset include a poor anastomotic technique, the use of woven sutures, a two-layer anastomosis, a long-gap EA, blood transfusion, anastomosis under tension and gastresophageal reflux [10]. Factors associated with mortality included a delayed diagnosis, premature birth, low birth weight and the presence of CHD. In the Spitz classification, patients with a birth weight of more than 1.5 kg are classified as a low-risk group; however, it is still possible to be classified as a group with high mortality and poor prognosis in the Waterston classification because of severe congenital malformations [11]. In our study, the incidence of anastomotic leak was 29.7%. We found that the birth weight of the anastomotic leak group was lower than that of the non-anastomotic leak group (2.61 ± 0.58 vs. 2.91 ± 0.47, *P* = 0.023). Nine of seventeen infants with CHD weighed less than 2.5 kg. Low birth weight is often associated with a history of premature delivery, foetal distress and heart malformation [12]. Low birth weight may be associated with poorer peripheral circulation and lower cardiac output, which may lead to a poor blood supply at the local anastomotic site. [13].

Survival is directly related to birth weight and CHD status. Infants weighing over 1500 g with no major cardiac problems had nearly 100% survival, whereas the presence of one risk factor reduced survival to 80%, and when both risk factors were present survival was further reduced to 30–50% [14]. Folaranmi indicated that the probability of esophageal anastomosis increased significantly with increasing body weight. His results showed that birth weight was a significant variable associated with the probability of primary esophageal anastomosis (OR = 1.009, *P* = 0.004) [15]. A study from Turkey also reported a significantly higher incidence of anastomotic leak in very-low-birth-weight infants compared with cases in the low- and normal-birth-weight groups [16]. Our study showed that CHD did not increase the risk of esophageal anastomotic leak (*P* = 0.721). This is consistent with the conclusions of Japanese scholars, who believe that CHD has nothing to do with anastomotic leak or stenosis [17]. Another study found no evidence that thoracoscopic repair of esophageal atresia impaired outcomes in children with congenital heart disease [18]. More interestingly, survival after treatment for EA was not influenced by the presence of, or the accuracy of, the diagnosis of CHD in this series. With only a few exceptions, associated CHD should not change the strategies of EA repair [19].

In clinical practice, we found that infants with anastomotic leak had lower perioperative albumin. Low albumin is an independent risk factor for postoperative anastomotic leak of the oesophagus [20]. A low patient albumin level preoperatively may result in postoperative anastomotic oedema, which also increases anastomotic tension. Preoperative albumin levels in the anastomotic leak group were noticeably lower than those in the non-anastomotic leak group. However, in multiple factor analysis, albumin levels were not included in the regression equation, which may be related to the fact that the number of cases with low albumin levels (albumin < 28 g/L) was lower. The sample size should be further expanded to find a more appropriate cut-off value to evaluate the relationship between albumin level and the incidence of anastomotic leak.

The treatment of long-gap EA is still difficult [21]. There is an increased risk of anastomotic leakage in long-gap EA. It usually requires extensive mobilization of the esophageal stump, which may impair the vascular supply to the oesophagus and consequently impair the healing ability of the anastomotic site [22]. Surgeons should carefully anastomose under low tension to prevent anastomotic complications during the initial repair of EA/TEF [23]. In this study, the average defect length was 1.52 ± 0.95 cm and 1.98 ± 1.08 cm in the anastomotic leak group and 1.33 ± 0.82 cm in the non-anastomotic leak group, and the difference was statistically significant. Serious anastomotic leak was found in 6 of the 8 patients with long-gap EA. A long gap is an independent risk factor for anastomotic leak. We believe that delayed anastomosis of a long segment defect may be a better choice, especially for infants with a gap length > 3 cm (intraoperative or preoperative), and the timing of anastomosis should be carefully determined.

Using the receiver operating curve, the AUC of this indicator was found to be 0.732, which had a good predictive value. We believe the index can be used as a good predictor of anastomotic leak during the perioperative period. For infants with a preoperative evaluation of the ratio of loss length to birth weight greater than 0.7, esophageal extension and gastrostomy should be considered. Anastomosis can be attempted after the natural extension of the oesophagus within the chest reaches a gap length of less than 3 cm, therefore, delayed repair may be a better choice [24].

As with any retrospective study, there is bias associated with data collection; this study was limited to one institution, and other institutions may have produced different results. A prospective study with a large group of patients and long-term follow-up is necessary.

## Conclusions

Birth weight and gap length are important predictors of anastomotic leak in EA. Our study shows that the ratio of gap length to birth weight index had a good predictive effect on the occurrence of anastomotic leak after anastomosis of EA.

## Data Availability

Data are available from the authors upon reasonable request.

## Abbreviations

EA: Esophageal atresia
TEF: Tracheal fistula
CHD: Congenital heart disease
OR: Odds ratio
95% CI: 95% Confidence intervals
ROC: Operating characteristic
AUC: Area under curve
VATS: Video-assisted thoracic surgery
